# Smartphone Photography as a Teledentistry Method to Evaluate Anterior Composite Restorations

**DOI:** 10.1155/2023/3171140

**Published:** 2023-07-11

**Authors:** Haleh Valizadeh-Haghi, Safa Valizadeh-Haghi, Nasim Naslseraji, Hamed Zandian

**Affiliations:** ^1^Department of Operative Dentistry, Ardabil University of Medical Sciences, Ardabil, Iran; ^2^Department of Operative Dentistry, Tabriz University of Medical Sciences, Tabriz, Iran; ^3^Social Determinants of Health Research Center, Ardabil University of Medical Sciences, Ardabil, Iran; ^4^Centre for Public Health and Wellbeing, School of Health and Social Wellbeing, College of Health, Science and Society, University of the West of England, Bristol, UK

## Abstract

**Objective:**

Today, teledentistry have gain more attention than past due to the advances in technology. The aim of this study was to compare the use of smartphone photography as a method in teledentistry with the face-to-face examination in the evaluation of anterior composite restorations.

**Materials and Methods:**

In this study, photographs of 24 composite restorations in patients attended to the clinic of Ardabil Dental School were obtained using a smartphone without any additional equipment and sent by email to 10 remote observers. As a gold standard method, these restorations were evaluated by an experienced expert in restorative dentistry a face-to-face examination. In both methods FDI criteria were used to evaluate the restorations and classified them as acceptable or not. Sensitivity, specificity, positive and negative predictive values, and diagnostic accuracy of the photographic method relative to face-to-face method were calculated. Furthermore the Mann–Whitney *U* test was used to statistically compare the two methods in detail.

**Results:**

The sensitivity, specificity, positive predictive value, and negative predictive value of the smartphone photography method was 69.35%, 48.72%, 87.34%, and 23.75%, respectively. A diagnostic accuracy of 65.97% was obtained. Statistically, in overall the photographic method rated the restorations as better than they were in reality (face-to-face examination) (*p* = 0.002).

**Conclusions:**

The diagnostic accuracy of the method of evaluating anterior composite restorations by smartphone photography was moderate and the use of this method in teledentistry, although was promising, needs to be improved.

## 1. Introduction

Today, advances in technology have affected various aspects of medicine like to the all aspects of life. These advances introduced the term teledentistry which is defined as a combination of telecommunication and dentistry [1]. Teledentistry has the potential to improve access to experts and services and to reduce the existing treatment costs and inequalities due to the differences in urban and suburban areas [2]. On the other hand, conditions such as the recent COVID-19 pandemic highlighted the need and importance of paying attention to this new tool in dentistry in order to further reduce the face-to-face visits [3]. Up to now, the use of photography in dentistry has increased, but still to a lesser extent has been used for diagnosis, consultation, and referral [4]. Studies have shown that photographic techniques in detecting oral diseases is comparable to the traditional visual methods [5, 6]. In a study of the ability of digital photography by intraoral camera to evaluate dental restorations it has been shown that this technique is a valid method, particularly in posterior restorations [7]. In a recent systematic review, it was concluded that despite evidence of the use and benefits of teledentistry in various aspects of dentistry, there is still a need for qualified studies to evaluate the effectiveness of this new tool [8].

It is clear that the quality of photographs and the equipment used to take them is so important as it can affect the accuracy of interpretation and analysis of photographs and may ultimately lead to suboptimal diagnosis [9]. The increasing use of smartphones and communication software in public has created a new era in the transfer of clinical data between patient and clinician [1]. Almost all smartphones have a camera and communication capability and are already available at a low cost and can be used as a method in teledentistry [10].

In the study by Estai et al. [10], photographs taken using a smartphone were acceptable compared to face-to-face examination in the diagnosis of dental caries. There are several promising studies and reports on the diagnosis of caries and oral lesions by the smartphone teledentistry [11–13]. However, there is no study that specifically examines restorations by smartphone photography. Today, resin composite is the material of choice in restoring anterior and posterior teeth [14]. Composite allows reproducing of the correct shade [15], translucency and anatomy of anterior teeth thus providing esthetic outcomes with conservative treatments [16]. Numerous studies have examined the clinical durability of composite restorations, indicating good clinical performance with an annual failure rate of 1.4%. However, replacing composite restorations still takes place and wastes a lot of time and money in health systems [17]. Anterior restorations have been shown to behave differently than posterior restorations in terms of causes of failure: recurrent caries is less pronounced in anterior restorations whereas other factors, including traumatic injuries, marginal wear due to the parafunction, and esthetic failures play more role in restoration replacement [18]. Considering these factors, and the lack of studies specifically about composite restorations, the aim of this study was to compare the evaluation of anterior composite restoration using smartphone photography with the face-to-face examination and to determine the diagnostic accuracy of this method. In this study, the FDI criteria were used for evaluating the restorations [19]. The null hypothesis of the study was that there is no difference between the two methods in evaluating anterior composite restorations.

## 2. Methods

This observational study conducted in Ardabil, Iran in 2020 in order to assess the diagnostic performance of smartphone photography as a tool for evaluating anterior composite restorations. Twenty-four anterior composite restorations in six patients were studied in a simple available sampling method in such a way that patients who attended to the Ardabil Dental School dentistry clinic in 2020 and had anterior composite restorations explained the objectives of the study and, if been a volunteer, participated in the study. The informed consent forms were completed by the participants and the study was ethically approved by ethics committee of Ardabil University of Medical Sciences under the code of IR.ARUMS.REC.1399.017. The sample size calculation was done using PS power and sample size calculation software based on estimating the difference in the mean overall scores as 1 between two groups, including the face-to-face and remote examination groups and aiming for a power of 90% and the *α* level of 0.05, which led to a sample size of 22. Finally, 24 restorations included in the study.

**Figure 1:**
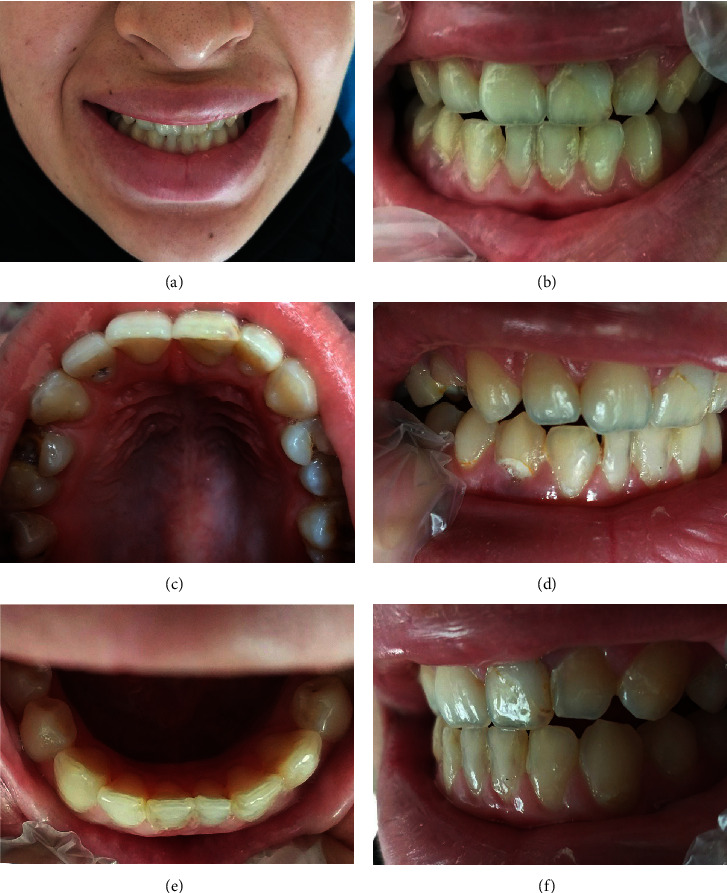
Example of photographs taken by smartphone camera: (a) smile, (b) frontal, (c) maxillary occlusal, (d) right occlusion, (e) mandibular occlusal, and (f) left occlusion views.

At first, the face-to-face examination of restorations was performed according to FDI criteria by an experienced specialist in restorative dentistry and the check lists were filled. The examination performed on the dental chair in the same single room with daylight at 10–12 am and was aided by mirrors, explorers, and the unit light if necessary. For the esthetic evaluation of restorations regarding shade matching, only daylight was used. This examination serves as the gold standard. Then the photographs of anterior teeth of patients were taken using a smartphone (Samsung Galaxy J8 (Dublin, IE)) with main dual camera of 16 and 5 MP resolution and LED flash. Six photographs in each patient were taken from frontal, right lateral, and left lateral occlusion, maxillary occlusal, mandibular occlusal, and smile view (Figure 1.) in the distance of approximately 2–5 cm. The photographs taken by a trained dental student without any equipment like the oral retractor or mirror. Only the daylight and the built-in flash of the smartphone's camera in auto-mode were used during the photo shoot.

The photographs were then emailed to 10 remote examiners which were specialists in restorative dentistry in order to assess the restorations and fill the FDI criteria checklists. Choosing 10 remote examiners was done based on a similar article [20], so we had 240 remote examinations of restorations. The standard FDI criteria are divided into three groups: esthetic parameters, functional parameters, and biological parameters. Each group of parameters includes a number of items or criteria and a score of 1–5 is assigned to each criterion as below:

1. Clinically excellent/very good
2. Clinically good
3. Clinically sufficient/satisfactory
4. Clinically unsatisfactory repair (but repairable)
5. Clinically poor (needs replacement).

The overall score of the restoration is determined from the scores of the subgroups so that the worst score is considered as the final score. Scores 1–3 are considered acceptable and scores 4 and 5 are considered unacceptable [19]. The original version of this criteria had 16 items, but because some items cannot be judged in photography, in the present study, a version by 12-items was used and the items of Radiographic examination, Patient's view, Postoperative (hyper-) sensitivity and tooth vitality, and Oral and general health were removed. An example of a filled form by remote participants provided in the Table 1. The filled forms collected and the final scores of each restoration per examines checked/calculated by a trained dental student as mentioned above.

Finally, in order to evaluate the accuracy of photographic method related to the face-to-face examination gold standard method sensitivity, specificity, positive and negative predictive values, and diagnostic accuracy were calculated according to the following formulas:

  Sensitivity = true positive/(true positive + false negative)
  Specificity = true negative/(true negative + false positive)
  Positive predictive value = true positive/(true positive + false positive)
  Negative predictive value = true negative/(true negative + false negative)
  Diagnostic accuracy = (true positive + true negative)/all subjects [21].

In addition to the above mentioned calculations, in order to statistically analyze the data, SPSS version 23 software (IMB, USA) was used. After examining the normal distribution of data by Kolmogorov–Smirnov test, the comparison of the two methods was done by Mann–Whitney *U* test in terms of each of the 12 items, three groups, and the overall result of the FDI criteria.

## 3. Results

The Kolmogorov–Smirnov test rejected the normal distribution of data, so the Mann–Whitney *U* test was used to analyze the hypotheses. The results of this test showed that the overall rating of the restorations were statistically different between the two methods studied (*p* = 0.002) and the average score was lower in the photographic method than the face-to-face method. The results also showed that in the case of esthetic, functional, and biological parameters with *p* = 0.2, *p* = 0.007, and *p* = 0.002, the same pattern exists between the photographic and face-to-face method. By further analysis of each of 12 items, it was found that there was no difference between the two methods in detecting surface luster (*p* = 0.18), staining (*p* = 0.9), color match and translucency (*p* = 0.57), esthetic anatomical form (*p* = 0.87), approximal anatomical form (*p* = 0.17), tooth integrity (*p* = 0.08), and adjacent mucosa (*p* = 0.06). However, in the diagnosis of fracture, the photographic method gave a worse score to the restorations (*p* = 0.02) than face-to-face examination but, it was less able to detect marginal adaptation problems (*p* = 0.007). The photographic method also gave a worse score (*p* = 0.03) in terms of occlusal contour and a better scores (*p* = 0.04) in the case of recurrent caries and periodontal response.

Considering the results of the photographic method in comparison with the gold standard of face-to-face examination (Table 2) and considering the prevalence of 83.61% of defective anterior composite restorations in the present study, diagnostic values were calculated for the photographic method, which shown in the Table 3.

## 4. Discussion

In the present study, a method of examining anterior composite restorations remotely by smartphone photography was assessed as a feasibility in teledentistry. The results showed that there is a statistical difference between the two methods used in this study, so the null hypothesis was rejected.

Among the various criteria for evaluating restorations, FDI criteria reported to be more discriminative with higher sensitivity than others, and it is said that it can detect the first signs of damage and failure. These criteria, introduced by Hickel et al. [19] were approved by the Science Committee of the FDI World Dental Federation in 2007 and were considered as the standard in 2008 and were highly recommended to be used in studies [22]. These criteria are a practical method with several criteria that can be selected based on the objectives of the study [19]. Accordingly, in the present study, 12 items that can be applicable to photographic analysis were selected and restorations were evaluated based on these criteria to accurately compare the two methods studied.

The statistical comparison showed that in terms of overall rating and three groups of esthetic, functional, and biological parameters of the criteria, the studied teledentistry method using smartphone photography resulted in evaluating the status of restorations as better than reality that confirmed by the diagnostic values as a moderate diagnostic accuracy was obtained for smartphone photography method. Furthermore, the specificity was less than the sensitivity and this shows that this method was weaker in detecting acceptable restorations than unacceptable ones. Additionally, considering the positive and negative predictive values, it can be said that more confidence can be placed in the positive reports (unacceptable restorations) of this method, and just about a quarter of reports as healthy restoration have been done correctly. All of these findings confirmed that the smartphone photography method had lower power in evaluating anterior composite restorations compared to gold standard. Since photography is a two-dimensional image that does not allow viewing all tooth surfaces, especially in the proximal areas, its limitations and the results of present study have been expected [12]. There are only few studies on the use of photography in the evaluation of restoration. In Signori's study, intraoral camera photography was reported as a valid method with high sensitivity and specificity for evaluating restorations, especially posterior restorations [7] in contrary to the results of the present study. It may be due to the use of an intraoral camera, a standardized photographic method and the use of a 50″ HD television and the same condition for photographic evaluations in that study [7] however, in the present study a simplified smartphone photography technique and uncontrolled end monitors characteristics for remote evaluations were used in order to assess the efficacy of a method that can be used everywhere. Furthermore, the evaluation of anterior restorations is probably more difficult than posterior restorations due to the higher subjectivity of esthetic factors which are more important for anterior restorations [7, 23]. In the study by Estai et al. [10], the sensitivity of the smartphone photography in the diagnosis of dental caries was moderate compared to the examination method, which was in line with the present study, but the high-specificity reported in that study was different from the low specificity of the present study. These differences can be attributed to the different methodologies and different equipment used in the studies. The quality of photographs taken by different equipment is different and affects the results of analyzes. Presence of saliva, debris, and unaided photography technique without retractor can be involved in low-quality photographs [24]. Intraoral cameras have been studied in the most studies. Despite the lower image quality and lower price than DSLR digital cameras, they are still not available in the remote areas and for lay persons. For this reason, smartphone photography has recently gained more attention due to its low price and weight, easy portability and availability, and no need to peculiar training to take photographs [9].

In the present study, a commonly used smartphone among the general public which was economically a medium level device was used and such an easy photographic protocol without assistance and special equipment was intentionally used to examine the effectiveness of this method as one that can be used by majority of nonprofessionals. However, all features of this smartphone like the quality of camera and LED internal flash used in the present study can influence the quality of images and the accuracy of remote diagnosis. Along with this, the transferring of images was done via email, and the monitor resolution and environmental conditions in which the remote examiners viewed the image could affect the results and all can be the subjects of future studies. However, according to the results of this study and similar studies, this method can be considered as a promising tool in large-scale screening and remote examination [10], or in situations such as the recent COVID-19 pandemic [3], but considering the moderate accuracy of the such simple method used in the present study, especially the low specificity, further studies and development of equipment to take more accurate photographs are highly recommended.

Based on the results of the present study, the photographic method had detected more cases of fracture and unproper occlusal anatomy, but was able to detect fewer problems in terms of marginal integrity, recurrent caries, and periodontal status. Similar studies are not available for direct comparison, but previous studies have reported more cases of defective restorations detected by intraoral camera photography [7] or more cases of fluorosis diagnosed by photography [25], which can be attributed to the possibility of image magnification to identify items that are neglected in clinical examination [26]. In contrast, the problem in distinguishing the true fracture line from the artifacts in photography can be related to reporting more cases of fracture [9]. In the case of poorer detection of marginal gaps, recurrent caries, and periodontal problems in the photography, it can be said that the use of mirror and explorer and better vision and tactile during clinical examination have been helpful in diagnosis, which could not be used in photography method. In a study on posterior restorations evaluated clinically and photographically, similar results were found about the diagnostic capability of the two methods regarding marginal integrity which explained by the importance of tactile examination by an explorer, however about recurrent caries they did not find significant differences between two methods despite present study [23]. It can be said that the orientation of anterior teeth can affect the visibility of different aspects of restorations in the photographs taken in the present study. Regarding periodontal health, previous studies concluded that photographic gingival color deviations usually occur by digital and intraoral cameras and can affect the accuracy of periodontal evaluation [27, 28]. On the other hand, even using scans with approximate true colors as Steinmeier et al. [20] used, periodontal conditions could not be assessed accurately in photographic evaluation so the importance of probing and clinical examination could not be ignored.

Finally, it should be said that further studies are needed about different ways to overcome the obstacles that exist in today's use of smartphone teledentistry. As concluded by previous studies these obstacles mainly are related to the difficulty of obtaining good photographs due to the lack of sufficient optimization of phone camera features, the absence of an oral retractor [24], the lack of training [24, 29], and some issues about laws and funding schemes [29]. The development of some proprietary applications to aid the proper orientation of dental arch for photo capturing, some kinds of commercial retractors designed for smartphone teledentistry and training dental educators, and mid-level health providers along with preparing some guidelines are the probable areas for future studies.

Despite the limitations of the present study, it can be concluded that the method of smartphone photography and sending images via email that used in the present study had moderate accuracy in evaluating the anterior composite restorations. Also, compared to face-to-face examination, the photographic method had a significant difference in terms of overall rating and esthetic, functional, and biological parameters and gave lower scores to the restorations.

## Figures and Tables

**Table 1:** The form used for the examinations. An example of filled one is demonstrated here.

Form is for: Patient A, upper left 2					
**Part A**					
A. Esthetic properties	1. Surface lustre	2. Staining a. Surface b. Margin	3. Color match and translucency	4. Esthetic anatomical form	

1. Clinically excellent/very good	1.1. Lustre comparable to enamel	*2a.1. No surface staining* *2b.1. No marginal staining*	3.1. Good color match, no difference in shade, and/or translucency	4.1. Form is ideal	

2. Clinically good (after polishing probably very good)	*1.2.1. Slightly dull, not noticeable from speaking distance* 1.2.2. Some isolated pores	2a.2. Minor surface staining, easily removable by polishing 2b.2. Minor marginal staining, easily removable by polishing	3.2. Minor deviations in shade and/or translucency	4.2. Form is only slightly deviated from the normal	

3. Clinically sufficient/satisfactory (minor shorcomings,no unacceptable effects but not adjustable w/o damage to the tooth)	1.3.1. Dull surface but acceptable if covered with film of saliva 1.3.2. Multiple pores on more than one third of the surface	2a.3. Moderate surface staining that may also present on other teeth, not esthetically unacceptable 2b.3. Moderate marginal staining, not esthetically unacceptable	3.3. Distinct deviation but acceptable. Does not affect esthetics: 3.3.1. More opaque 3.3.2. More translucent 3.3.3. Darker 3.3.4. Brighter	4.3. Form deviates from the normal but is esthetically acceptable	

4. Clinically unsatisfactory (but reparable)	1.4.1. Rough surface, cannot be masked by saliva film, simple polishing is not sufficient. Further intervention necessary 1.4.2. Voids	2a.4. Unacceptable surface staining on the restoration and major intervention necessary for improvement 2b.4. Pronounced marginal staining; major intervention necessary for improvement	3.4. Localized clinically deviation that can be corrected by repair: 3.4.1. Too opaque 3.4.2. Too translucent 3.4.3. Too dark 3.4.4. Too bright	4.4. Form is affected and unacceptable esthetically. Intervention/correction is necessary	

*5. Clinicaly poor* *(replacement necessary)*	1.5. Very rough, unacceptable plaque retentive surface	2a.5. Severe surface staining and/or subsurface staining, generalized, or localized, not accessible for intervention. 2b.5. Deep marginal staining, not accessible for intervention	*3.5. Unacceptable replacement necessary*	*4.5. Form is unsatisfactory and/or lost. Repair not feasible/reasonable, replacement needed*	
**Part B**					

B. Functional properties	5. Fracture of material and retention	6. Marginal adaptation	7. Occlusal contour and wear (a) Qualitatively (b) Quantitatively	8. Approximal anatomical form (a) Contact point (b) Contour	

1. Clinically excellent/very good	5.1. No fractures/cracks	6.1. Harmonious outline, no gaps, no white, or discolored lines	*7a.1. Physiological wear equivalent of enamel* *7b.1. Wear corresponding to 80%–120% of enamel*	*8a.1. Normal contact point (floss or 25 µm metal blade can pass)* *8b.1. Normal contour*	

*2. Clinically good*	*5.2. Small hairline crack*	6.2.1. Marginal gap (<150 *µ*m), white lines 6.2.2. Small marginal fracture removable by polishing *6.2.3. Slight ditching, slight step/flashes, minor irregularities*	7a.2. Normal wear only slightly different from that to enamel 7b.2. 50%–80% or 120%–150% wear compared to that of corresponding enamel	8a.2. Contact slightly too strong but no disadvantage (floss or 25 *µ*m metal blade can only pass with pressure) 8b.2. Slightly deficient contour	

3. Clinically sufficient/satisfactory (minor shortcomings, no unacceptable effects but not adjustable w/o damage to the tooth)	5.3. Two or more or larger hairline cracks and/or material chip fracture not affecting the marginal integrity or approximal contact	6.3.1. Gap < 250 *µ*m not removable 6.3.2. Several small marginal fractures 6.3.3. Major irregularities, ditching, or flash, steps	7a.3. Different wear rate than enamel but within the biological variation 7b.3. < 50% or 150%–300% of corresponding enamel	8a.3. Somewhat weak contact, no indication of damage to tooth, gingiva or periodontal structures; 50 *µ*m metal blade can pass 8b.3. Visible deficient contour	

4. Clinically unsatisfactory/(but reparable	5.4.1. Material chip fractures which damage marginal quality or approximal contacts 5.4.2. Bulk fractures with partial loss (less than half of the restoration)	6.4.1. Gap >250 *µ*m or dentin/base exposed 6.4.2. Severe ditching or marginal fractures 6.4.3. Larger irregularities or steps (repair necessary)	7a.4. Wear considerably exceeds normal enamel wear; or occlusal contact points are lost 7b.4. Restoration >300% of enamel wear or antagonist >300%	8a.4. Too weak and possible damage due to food impaction; 100 *µ*m metal blade can pass 8b.4. Inadequate contour repair possible	

5. Clinicaly Poor (replacement necessary)	5.5. (Partial or complete) loss of restoration or multiple fractures	6.5.1. Restoration (complete or partial) is loose but in situ. 6.5.2. Generalized major gaps or irregularities	7a.5. Wear is excessive 7b.5. Restoration or antagonist >500% of corresponding enamel	8a.5. Too weak and/or clear damage due to food impaction and/or pain/gingivitis 8b.5. Insufficient contour requires replacement	
**Part C**					

C. Biological properties	9. Recurrence of caries (CAR), erosion, abfraction	10. Tooth integrity (enamel cracks, tooth fractures)	11. Periodontal response (always compared to a reference tooth)	12. Adjacent mucosa	

1. *Clinically very good*	9.1. *No secondary or primary caries*	10.1. *Complete integrity*	11.1. *No plaque, no inflammation, no pockets*	12.1. *Healthy mucosa adjacent to restoration*	

2. Clinically good (after correction maybe very good) no treatment required	9.2. Small and localized (1) Demineralization (2) Erosion or (3) Abfraction	10.2.1. Small marginal enamel fracture (<150 *µ*m) 10.2.2. Hairline crack in enamel (<150 *µ*m)	11.2. Little plaque, no inflammation (gingivitis), no pocket development 11.2.1. Without 11.2.2. With overhangs, gaps or inadequate anatomic form	12.2. Healthy after minor removal of mechanical irritations (plaque, calculus, sharp edges, etc.)	

3. Clinically sufficient/satisfactory (minor shortcomings with no adverse effects but not adjustable without damage to the tooth)	9.3. Larger areas of (1) Demineralisation, (2) Erosion, or (3) Abrasion/abfraction, dentin not exposed Only preventive measures necessary	10.3.1. Marginal enamel defect <250 *µ*m 10.3.2. Crack <250 *µ*m; 10.3.3. Enamel chipping 13.3.4 multiple cracks	11.3. Difference up to one grade in severity of PBI compared to baseline and compared to control tooth 11.3.1. Without 11.3.2. With overhangs, gaps or inadequate anatomic form	12.3. Alteration of mucosa but no suspicion of causal relationship with restorative material	

4. Clinically unsatisfactory (repair for prophylactic reasons)	9.4.1. Caries with cavitation and suspected undermining caries 9.4.2. Erosion in dentin 9.4.3. Abrasion/abfraction in dentin Localized and accessible can be repaired	10.4.1. Major marginal enamel defects; gap > 250 *µ*m or dentin or base exposed 10.4.2. Large cracks >250 *µ*m, probe penetrates 10.4.3. Large enamel chipping or wall fracture	11.4. Difference of more than one grade of PBI in comparison to control tooth or increase in pocket depth >1 mm requiring intervention. 11.4.1. Without 11.4.2. With overhangs, gaps, or inadequate anatomic form	12.4. Suspected mild allergic, lichenoid, or toxic reaction	

5. Clinically poor (replacement necessary)	9.5. Deep caries or exposed dentin that is not accessible for repair of restoration	10.5. Cusp or tooth fracture	11.5. Severe/acute gingivitis or periodontitis 11.5.1. Without 11.5.2. With overhangs, gaps, or inadequate anatomic form	12.5. Suspected severe allergic, lichenoid, or toxic reaction	
Result:					

Scores	Acceptable			Unacceptable	
	1	2	3	4	5

Esthetic properties					^*∗*^

Functional properties		^*∗*^			

Biological properties	^*∗*^				

Overall score					^*∗*^

Italic values were the examples of filled form and were important.

**Table 2:** Results of the smartphone photography method and the gold standard method of face-to-face examination.

Test results of^†^		Gold standard		Total
		+	−	
Smartphone photography	^+^	138	20	158
	^−^	61	19	80

Total		199	39	238

^†^Unacceptable restorations are considered as a positive test result.

**Table 3:** Measures of diagnostic accuracy of smartphone photography method in evaluating anterior composite restorations.

Measures	(%)
Sensitivity	69.35
Specificity	48.725
Positive predictive value	87.34
Negative predictive value	23.75
Diagnostic accuracy	65.97

## Data Availability

Data are available on request via email to corresponding author.
